# Primary Diffuse Leptomeningeal Gliomatosis: Radiological/Pathological Features

**DOI:** 10.1155/2016/5016840

**Published:** 2016-11-07

**Authors:** Ehtasham Ahmad, Mohamed Mohamed, Apostolos Vrettos

**Affiliations:** ^1^East Kent Hospitals University NHS Trust, Queen Elizabeth the Queen Mother Hospital, Department of General Internal Medicine, Margate, UK; ^2^Teesside University, School of Health & Social Care, Middlesbrough, UK

## Abstract

We present the case of a 43-year-old lady who presented with headaches, visual impairment, and seizures, progressing rapidly over the course of a few weeks. Extensive workup excluded an inflammatory or infectious cause. Imaging studies revealed diffuse thickening of the leptomeninges and serial CSF analysis showed raised opening pressures and increased protein levels. A diagnostic biopsy of the lower thoracic dura confirmed the diagnosis of primary diffuse leptomeningeal gliomatosis (PDGL). She was managed supportively for her symptoms and unfortunately she passed away a few weeks later.

## 1. Introduction

Primary diffuse leptomeningeal gliomatosis is a rare cause of raised intracranial pressure and can affect any patients of any age. It is a challenging diagnosis requiring expert opinion and other differentials must be ruled out before reaching the final diagnosis which usually can only be confirmed with a biopsy.

## 2. Case Report

A 43-year-old lady presented to emergency department with a 3-week history of intermittent headaches, deteriorating vision, and dizziness with nausea and vomiting. A few days later she started having episodes of generalized tonic-clonic fits. There was no history of fever, weight loss, or recent travel abroad. Her past medical history was significant for an episode of left sided weakness with facial weakness at age 19 which resolved on its own with no residual deficit. A CT scan at that time showed an area of damage in the right hemisphere within the white matter; these appearances were suggestive of an infarct. An MRI scan done at that time did not show any features consistent with multiple sclerosis. CSF analysis was within normal limits. Visual evoked potentials were also normal. The exact cause for this episode was not found and the patient was discharged with the advice to quit smoking. No further tests were done at that time. Since then, she remained fit and healthy with no further episodes of weakness. She was not on any regular medications. Her family history was unremarkable. Physical examination, including a full neurological examination, was also unremarkable. There was no focal neurology, neck stiffness, or any other signs of meningism.

**Figure 1:**
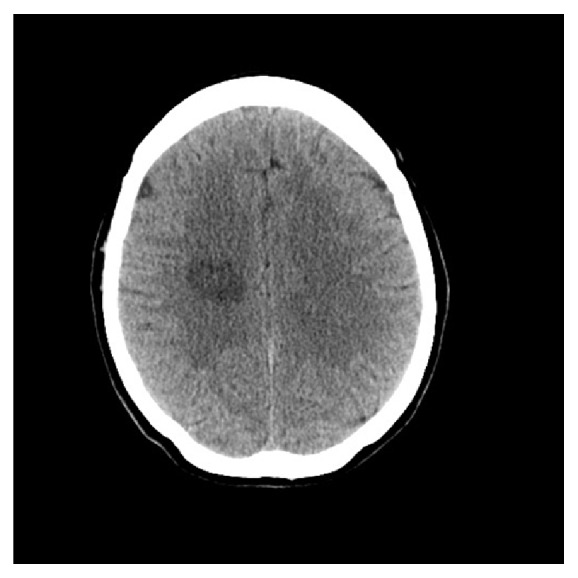
Axial head CT.

**Figure 2:**
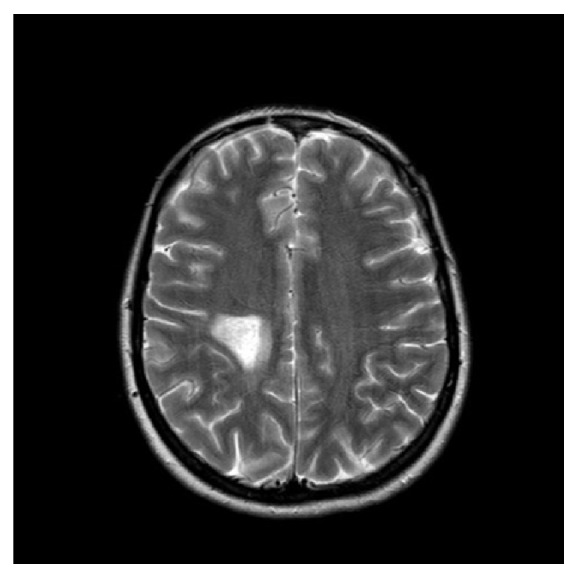
Axial head MRI.

**Figure 3:**
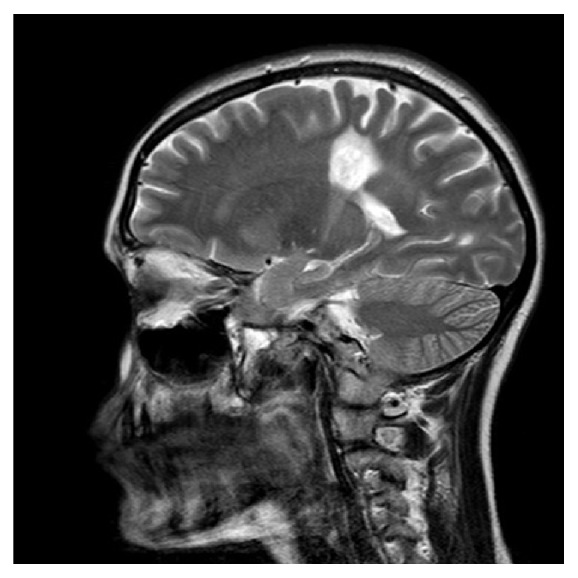
Sagittal head MRI.

Routine lab tests were all normal. With this presentation, head CT scan was an obvious first choice investigation. It showed an area of low density within the right parietal region measuring about 2.7 cm, with no evidence of enhancement following injection of IV contrast and no mass effect (Figure 1). A head MRI scan showed mature damage most likely due to an old infarct (Figure 2) but it also revealed leptomeningeal enhancement along several regions of the brain (Figure 3).

**Figure 4:**
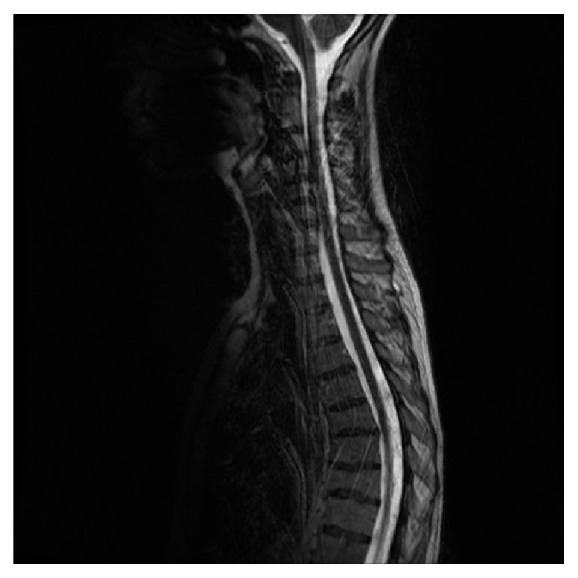
Spine MRI, thoracic region.

**Figure 5:**
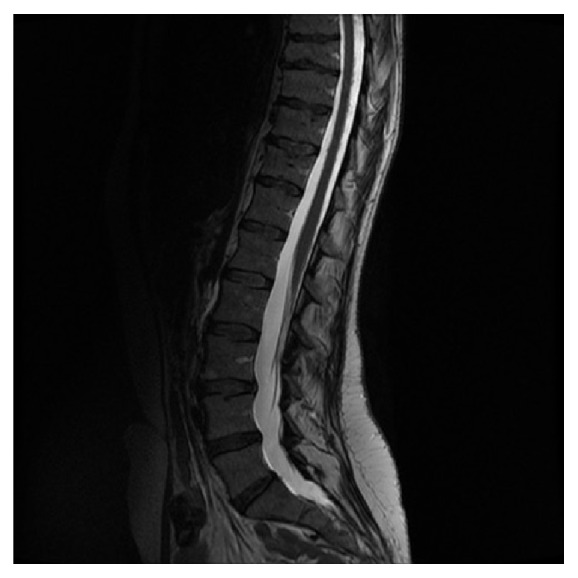
Spine MRI, lumbosacral region.

Based on the findings of the head CT scan and head MRI scan the differentials included diffuse leptomeningeal disease (with an old infarct) as a result of inflammation secondary to sarcoidosis, infection with tuberculosis (TB), and malignancy either primary or as a result of an underlying metastatic process from lung or breast. She then had a chest X-ray and then a CT scan of the chest and abdomen which did not show any evidence of an underlying malignancy. A complete vasculitic screen, including ANCA antibodies, was negative. Serology for syphilis, HIV, HBV, and HCV was all negative. The images were then transferred to a tertiary centre (Walton Neurology Centre in Liverpool) for further review by a specialist neuroradiologist and expert opinion was sought from a neurologist. His opinion was that the top differential could be neurosarcoidosis and he recommended starting her on steroids and doing a lumbar puncture for CSF analysis. The lumbar puncture showed increased opening pressure of 41 cm of H_2_O. The CSF analysis revealed cellular deposit containing a mixed population of small lymphoid cells with no malignant cells. Also, on the CSF analysis, there was increased protein content of 12,90 g/L. A CSF culture was negative for TB. ACE levels and serum calcium were requested and both came back as normal, but the neurologist suggested continuing with the steroids as the patient reported some improvement of her symptoms. More specifically, she reported some transient improvement in her visual symptoms and there was also a decrease in the CSF opening pressure on a repeated lumbar puncture. In terms of other differentials, lymphoma was low on the probability as there was no history of weight loss, fever, or lymphadenopathy. Serum LDH was also within the normal range. Fundoscopy did not show any evidence of papilloedema. 2-3 weeks after being started on steroids, her vision continued to deteriorate. She became totally blind in the left eye and her vision was reduced to light perception in the right eye. Serial CSF analysis continued to show raised opening pressure with high CSF protein but the neurology multidisciplinary team decided not to do any shunting as the shunt could be blocked by the high CSF protein content. An MRI scan of her spine showed diffuse meningeal enhancement coating the whole of the spinal cord down to the conus and extending to cover the nerve roots of the cauda equina. The cord itself returned a normal signal (Figures 4 and 5).

**Figure 6:**
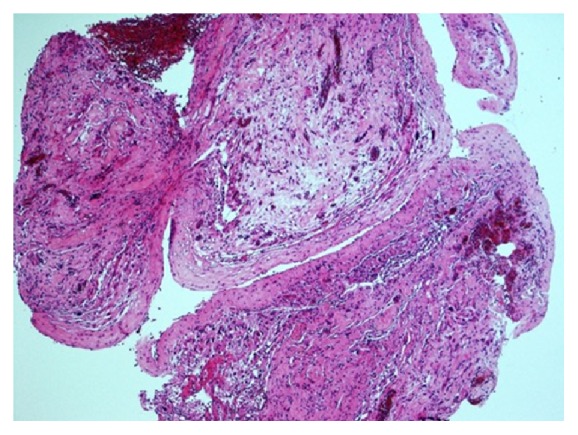
The biopsy from the lower thoracic dura showed fibrous tissue lined by meningothelial type of cells with nests of moderately pleomorphic cells. These cells appeared fibrillary, rounded, and occasionally gemistocytic or multinucleated. Proliferating vascular clusters were present as well. Occasional round cells were seen forming clusters in plane.

To reach the final diagnosis a biopsy was taken from the lower thoracic dura (Figure 6). The appearances of the biopsy were in keeping with a diagnosis of leptomeningeal gliomatosis. Three previous CSF cytospin preparations did not show any atypical cells. Immunohistochemical staining showed that the cell nests were GFAP and S100 positive. A few scattered proliferating nuclei were present (Ki67). A few scattered LCA positive lymphocytes were seen. The following markers were negative: CAM5.2 and IDH1.

By the time final diagnosis was reached the patient's symptoms had already progressed considerably. More specifically her visual impairment progressed to complete blindness over the next few weeks and she also developed weakness in both lower limbs with a motor power of 1-2/5 bilaterally. Sphincter function was also lost. Around the time of her death she had flaccid paralysis and unresponsive plantar reflexes. She did not respond to steroids any more but steroids were continued up until a few days before her death. Once the diagnosis was established, along with the prognosis, which happened about one week before her death, the steroids were stopped as they were not making any difference. After consultation with her family, it was decided to treat her palliatively. She was referred to the palliative care team and unfortunately she passed away in the next few days, after a total of 4 months since she first presented. The cause of death was due to aspiration pneumonia and a postmortem examination was not performed.

## 3. Discussion

Primary diffuse leptomeningeal gliomatosis is a rare condition characterised by infiltration of the meninges by neoplastic glial cells without evidence of primary tumour in the brain or the spinal cord [1]. It is an ultimately fatal condition which is characterised by infiltration of meninges by tumour cells composed of nests of heterotopic glial cells. Glial heterotopia is defined as nests or linear arrays of glial tissue in the meninges [2]. Primary leptomeningeal glioma can be in the form of a single solitary tumour or a diffuse tumour involving the intracranial or the spinal cord's leptomeninges [3]. The usual presentation includes symptoms and signs of raised intracranial pressure such as headache, nausea, vomiting, seizures, and papilloedema, and, based on a review of similar reported cases, it has been suggested that the presence of raised intracranial pressure with marked leptomeningeal enhancement on imaging should raise the suspicion of PDLG [3]. The diagnosis relies mainly on the biopsy of meningeal tissue. For this reason, antemortem diagnosis is a challenge and approximately one-third of cases are diagnosed on postmortem examination [3]. Debono et al. have proposed the following diagnostic criteria [4]:

1. No apparent attachment of extramedullary meningeal tumour to the neural parenchyma.
2. No evidence of primary neoplasia within the neuraxis.
3. The existence of distinct leptomeningeal encapsulation around the tumour.

A common finding on the imaging studies is the focal or diffuse leptomeningeal enhancement with contrast. The CSF analysis usually demonstrates raised opening pressure with raised protein count, as in our patient, with pleocytosis. The CSF can rarely show red blood cells as well [5]. Cytology for malignant cells is usually negative and for this reason it is advised to do GFAP staining in CSF cytospin to detect cells of glial origin [6]. There is no definitive cure for this ultimately fatal condition. The treatment mainly relies on the relief of the symptoms. Ventriculoperitoneal shunting is sometimes used to decrease the raised intracranial pressure [7]. Peritoneal tumour dissemination through VP shunt has not been reported in PDGL [2]. In this case, this lady received steroids, for presumed neurosarcoidosis. Interestingly, this incidentally led to some temporary improvement of her symptoms, a finding also reported in a similar case of PDGL [8]. The prognosis is poor with a mean survival time after diagnosis of approximately 4 months. Only a few cases have been reported to have responded to treatment [9–11] and the longest survival has been reported with the combination of radiotherapy and temozolomide [12, 13].

## Additional Points

*Learning Points.* (i) Primary diffuse leptomeningeal gliomatosis is a rare malignancy involving meningeal tissue without involving the brain or spinal cord parenchyma. (ii) The patients usually present with signs and symptoms of raised intracranial pressure. The presence of raised intracranial pressure with marked leptomeningeal enhancement on imaging should raise the suspicion of PDLG. (iii) Definite diagnosis requires biopsy.

## References

[1] Ruiz-Ares G., Collantes-Bellido E., Rodriguez De Rivera F. (2011). Primary diffuse leptomeningeal gliomatosis mimicking meningeal tuberculosis. *Neurologist*.

[2] Bilic M., Welsh C. T., Rumboldt Z., Hoda R. S. (2005). Disseminated primary diffuse leptomeningeal gliomatosis: a case report with liquid based and conventional smear cytology. *CytoJournal*.

[3] Jabeen S. A., Chowdary A. H., Kandadai R. M. (2014). Primary diffuse leptomeningeal gliomatosis: an autopsy case report. *Annals of Indian Academy of Neurology*.

[4] Debono B., Derrey S., Rabehenoina C., Proust F., Freger P., Laquerrière A. (2006). Primary diffuse multinodular leptomeningeal gliomatosis: case report and review of the literature. *Surgical Neurology*.

[5] Dietrich P.-Y., Aapro M. S., Rieder A., Pizzolato G. R. (1993). Primary diffuse leptomeningeal gliomatosis (PDLG): a neoplastic cause of chronic meningitis. *Journal of Neuro-Oncology*.

[6] Wechsler L. R., Gross R. A., Miller D. C. (1984). Meningeal gliomatosis with ‘negative’ CSF cytology: the value of GFAP staining. *Neurology*.

[7] Beauchesne P., Pialat J., Duthel R. (1998). Aggressive treatment with complete remission in primary diffuse leptomeningeal gliomatosis. A case report. *Journal of Neuro-Oncology*.

[8] Ko M. W., Turkeltaub P. E., Lee E. B. (2009). Primary diffuse leptomeningeal gliomatosis mimicking a chronic inflammatory meningitis. *Journal of the Neurological Sciences*.

[9] Beauchesne P., Pialat J., Duthel R. (1998). Aggressive treatment with complete remission in primary diffuse leptomeningeal gliomatosis: a case report. *Journal of Neuro-Oncology*.

[10] Gonçalves A. L., Masruha M. R., Carrete H., Stávale J. N., Da Silva N. S., Vilanova L. C. P. (2008). Primary diffuse leptomeningeal gliomatosis. *Arquivos de Neuro-Psiquiatria*.

[11] Hansen N., Wittig A., Hense J., Kastrup O., Gizewski E. R., Van De Nes J. A. P. (2011). Long survival of primary diffuse leptomeningeal gliomatosis following radiotherapy and temozolomide: case report and literature review. *European Journal of Medical Research*.

[12] Dorner L., Fritsch M. J., Hugo H. H., Mehdorn H. M. (2009). Primary diffuse leptomeningeal gliomatosis in a 2 year-old girl. *Surgical Neurology*.

[13] Hansen N., Wittig A., Hense J., Kastrup O., Gizewski E. R., Van de Nes J. A. P. (2011). Long survival of primary diffuse leptomeningeal gliomatosis following radiotherapy and temozolomide: case report and literature review. *European Journal of Medical Research*.

